# Treatment, transport, and primary care involvement when helicopter emergency medical services are inaccessible: a retrospective study

**DOI:** 10.1080/02813432.2018.1523992

**Published:** 2018-10-08

**Authors:** Dag Ståle Nystøyl, Steinar Hunskaar, Hans Johan Breidablik, Øyvind Østerås, Erik Zakariassen

**Affiliations:** a Department of Research, Norwegian Air Ambulance Foundation, Drøbak, Norway;; b Research Group for General Practice, Department of Global Public Health and Primary Care, University of Bergen, Bergen, Norway;; c National Centre for Emergency Primary Health Care, Uni Research Health, Bergen, Norway;; d Centre of Health Research, Førde Hospital Trust, Førde, Norway;; e Department of Anaesthesia and Intensive Care, Haukeland University Hospital, Bergen, Norway;; f Department of Clinical Medicine, Faculty of Medicine and Dentistry, University of Bergen, Bergen, Norway

**Keywords:** Emergency medical services, primary health care, air ambulances, Norway, HEMS, general practitioners

## Abstract

**Objective:** To examine handling of cancelled helicopter emergency medical services (HEMS) missions with a persisting medical indication.

**Design:** Retrospective observational study.

**Setting and subjects:** Cancelled HEMS missions with persisting medical indication within Sogn og Fjordane county in Norway during the period of 2010–2013. Both primary and secondary missions were included.

**Main outcome measures:** Primary care involvement, treatment and cooperation within the prehospital system.

**Results:** Our analysis included 172 missions with 180 patients. Two-thirds of the patients (118/180) were from primary missions. In 95% (112/118) of primary missions, GPs were alerted, and they examined 62% (70/112) of these patients. Among the patients examined by a GP, 30% (21/70) were accompanied by a GP during transport to hospital. GP involvement did not differ according to time of day (*p* = 0.601), diagnostic group (*p* = 0.309), or patient’s age (*p* = 0.409). In 41% of primary missions, the patients received no treatment or oxygen only during transport. Among the secondary missions, 10% (6/62) of patients were intubated or received non-invasive ventilation and were accompanied by a physician or nurse anaesthetist during transport.

**Conclusions:** Ambulance workers and GPs have an important role when HEMS is unavailable. Our findings indicated good collaboration among the prehospital personnel. Many of the patients were provided minimal or no treatment, and treatment did not differ according to GP involvement.

**Key Points**
- Knowledge about handling and involvement of prehospital services in cancelled helicopter emergency medical services (HEMS) missions are scarce.
- Ambulance workers and general practitioners have an important role when HEMS is unavailable
- Minimal or no treatment was given to a large amount of the patients, regardless of which health personnel who encountered the patient.

## Introduction

On-call general practitioners (GPs) and the ambulance service constitute the backbone of the pre-hospital emergency medical service (EMS) in Norway. GPs regularly complete re-training in emergency medicine. Ambulance workers complete at least two years of upper secondary school and two years as an apprentice [1]. Every municipality in Norway is obligated to have a doctor on call around the clock, who can potentially call out immediately in emergencies [2]. Many OOH services are inter-municipal co-operations. GPs and the ambulance service handle the majority of medical emergencies without requiring helicopter emergency medical services (HEMS). Involvement of GPs in emergency patients differs between European countries, i.e. Denmark where GPs perform telephone triage and anaesthesiologists encounter patients outside hospital [3–5].

HEMS have been an integrated part of the Norwegian EMS since 1988. The main indication for HEMS is severe disease or trauma requiring rapid transport and/or advanced triage, treatment, and supervision. National guidelines advise the use of HEMS when this is anticipated to improve health outcome compared to the use of ground ambulance [6]. Norway has 13 helicopters and 6 search-and rescue (SAR) helicopters staffed with anaesthesiologists. The goal of being able to reach 90% of the population within 45 minutes is achieved on a national level, but with some differences between the HEMS bases [7].

To ensure equal access to public health services regardless of residence, the EMS must be sustainable in all areas and seasons, even under challenging weather conditions. Over the last decade, ambulances and OOH services have been centralized such that they now cover larger geographical areas, resulting in longer response times [8,9]. In this context, HEMS may serve to compensate for potentially unequal access to emergency medical care. However, the advantages of HEMS are controversial, with previous studies showing inconsistent results regarding the benefits for patients [10–18]. A Cochrane review concluded that it is unclear which elements provided by HEMS benefit the patients [19].

In 2014, 38% of all HEMS requests in Norway were cancelled. The main reason for cancellation was that there was no longer a medical indication (20%). Other reasons for cancellation included weather conditions (9%), concurrency conflicts (5%) and other (4%) [20]. Scarce data are available regarding the alternatively handling of patients for HEMS missions that are cancelled despite persisting medical indication. Such knowledge is important for the development of an optimally organized EMS.

In the present study, we investigated HEMS missions that were cancelled for non-medical reasons, with the aims of determining the extent of primary care involvement, the treatment provided, and the cooperation between the prehospital services.

## Methods

### Design and study setting

We designed a retrospective observational study to investigate the aims. The study area was the county of Sogn og Fjordane (S&F), located in the western part of Norway and has a challenging topography with fjords, islands, and high mountains. The total area is 18,623 km^2^ and the county is sparsely populated with a total of 109,000 inhabitants (2013). Rough weather conditions present a challenge for HEMS and ground ambulances. The county has three hospitals. One is located in Førde, and admits patients with emergency internal medical and surgical needs. The other two are located in Nordfjordeid and Laerdal, and each treats emergency internal medical conditions and has an on-call anaesthesiologist. Patients suffering major trauma and/or severe head injury, and patients requiring PCI or thrombectomy are transported to Haukeland University Hospital in Bergen. One HEMS base is located in Førde, and one SAR helicopter is located in Florø, which is approximately 45 minutes by road from Førde. Four other HEMS bases are located in the neighbouring counties, and can be alerted when HEMS Førde is unavailable. These helicopters can also reach a majority of the population of S&F county within 45 minutes.

At the time of this study, S&F county had 21 ground ambulance stations and 15 OOH services. Within the largest OOH service area, it can take up to two hours of driving time for the on-call GP to reach a patient. Ground ambulances have often shorter driving time to the patients, compared to the on-call GP, and ambulance workers can in some situations perform protocol-based treatment, such as administration of morphine, oxygen, nitroglycerine, and acetylsalicylic acid (MONA), without physician involvement.

At all Emergency Medical Commination Centers (EMCC) in Norway, operators with emergency care experience use the Norwegian Index for Emergency Assistance (Index) as dispatch guidelines for determining mission urgency and the appropriate level of response [21,22]. The Index is a symptom-based criteria system that includes three response levels: acute, urgent and non-urgent. Based on the information provided and the Index criterion, the operator determines whether HEMS should be dispatched. Subsequently, the HEMS anaesthesiologist evaluates whether there is a medical indication for HEMS, and the pilot decides if the weather conditions are acceptable.

### Materials

For this study, we evaluated emergency missions in S&F county for which HEMS were requested, during the period from January 1, 2010 to December 31, 2013. Our analyses included all events where HEMS had to cancel the mission for non-medical reasons, including weather conditions, technical reasons, exceeded duty time, or concurrencies. Both primary (on-scene) and secondary (inter-hospital) missions were included. SAR data were available for 2012–2013. The inclusion criteria allowed for patient inclusion independent of clinical condition.

In S&F county, HEMS register mission and patient data in the AirDoc database. Each dispatch in AirDoc has a unique identification number generated from the Acute Medical Information System (AMIS), a database in which the EMCC in Førde reports activity and mission data. AMIS contains national person identification numbers that makes it possible to access and link data from other records.

Cancelled missions were identified in AirDoc. AMIS identification numbers were extracted from AirDoc. We then obtained mission data from AMIS, including: Age, gender, date, Index criteria code, resources alarmed, response to alarm, timeline for each resource, site, destination and a free text field. Ambulance records contained clinical data about the patients, treatment provided and which health personnel being involved. Records from hospitals and OOH services were retrieved to supplement missing data, such as inconsistent information from AMIS or data about treatment. All data were collected retrospectively. Diagnostic group categorization was performed by the researchers (EZ and DSN) and was based on the assumed medical problem at the time of requesting HEMS, using both Index code and free text information. Cooperation was defined as presence at the scene, assuming communication and involvement between health personnel.

### Statistical analysis

Standard descriptive analyses were performed using SPSS Statistics Version 22/23 (IBM Corp., Armonk, NY, USA). Due to skewed data, age is presented as median with interquartile range (IQR). Pearson’s Chi-square and Fischer’s exact test were used for categorical variables, and *p* values of <0.05 were considered statistically significant. Table 1 is stratified for primary and secondary missions. GP involvement vs GP not involved was compared in Table 2. Only treatments in primary missions are showed in Table 2, due to minimal treatments given in secondary missions.

**Table 1.** Demographic and mission data from 180 cases for which HEMS was unavailable. Stratification by primary and secondary missions^a^.

	All patients		Patients from primary mission		Patients from secondary mission	
	*N* = 180		*N* = 118		*N* = 62	
Gender	*N*	(%)	*N*	(%)	*N*	(%)
Female	71	(39)	47	(40)	24	(39)
Median age	Years	IQR	Years	IQR	Years	IQR
Male	56	32–72	54	38–71	62	21–73
Female	61	30–72	64	31–73	58	16–67
Total	59	31–72	57	36–72	60	20–71
Diagnostic group	*N*	(%)	*N*	(%)	*N*	(%)
Cardiology	63	(35)	46	(39)	17	(27)
Neurology	34	(19)	28	(24)	6	(10)
Surgery	10	(6)	2	(2)	8	(13)
Infection	13	(7)	3	(3)	10	(16)
Breathing difficulties	7	(4)	6	(5)	1	(1)
Obstetric	12	(7)	2	(2)	10	(16)
Intoxication	3	(1)	2	(2)	1	(1)
Trauma	27	(15)	23	(19)	4	(7)
Other	11	(6)	6	(5)	5	(8)
Site/Scene	*N*	(%)	*N*	(%)	*N*	(%)
Home dwelling	76	(42)	76	(64)	0	(0)
Public area	29	(16)	29	(25)	0	(0)
Casualty clinic/Nursing home	11	(6)	11	(9)	0	(0)
Local hospital	35	(19)	0	(0)	35	(57)
County hospital	27	(15)	0	(0)	27	(43)
Other	2	(1)	2	(2)	0	(0)
Destination	*N*	(%)	*N*	(%)	*N*	(%)
Discharged on-scene	3	(2)	3	(3)	0	(0)
Dead on-scene	5	(3)	5	(4)	0	(0)
Casualty clinic	5	(3)	5	(4)	0	(0)
Local hospital	33	(18)	33	(27)	0	(0)
County hospital	74	(41)	57	(48)	17	(27)
University hospital	52	(29)	15	(13)	37	(60)
Remain in hospital	8	(4)	0	(0)	8	(13)
Supervision by health personnel during transport^b^	*N*	(%)	*N*	(%)	*N*	(%)
Ambulance workers only	121	(67)	94	(80)	27	(44)
+General practitioner	21	(12)	21	(18)	0	(0)
+Anaesthesiologist	10	(6)	1^c^	(1)	9	(15)
+Midwife	8	(4)	1	(1)	7	(11)
+Nurse anaesthetist	7	(4)	0	(0)	7	(11)
+Hospital physician	1	(1)	0	(0)	1	(1)
+Other	3	(2)	1	(1)	2	(3)
Missing	9	(5)	0	(0)	9	(15)

^a^Primary mission: patient outside hospital. Secondary mission: patient in need of interhospital transport

^b^Highest level of health personnel in contact with the patient during transport. Ranking: Anesthesiologist, GP/hospital physician, nurse anaesthetist/midwife and other.

^c^Mission during which an anaesthesiologist from the hospital called out to a patient living near the hospital.

**Table 2.** Treatment provided on primary missions according to general practitioner (GP) response and involvement.

	GP response on primary missions (*N* = 118)						
	GP involvement						
	On site^a^ (*N* = 70)		Conferred with ambulance (*N* = 13)		GP not involved (*N* = 35)		*p* Value
Treatment	*n*	%	*n*	%	*n*	%	
No treatment	17	(24)	3	(23)	11	(31)	0.490
Oxygen only	11	(16)	2	(15)	4	(11)	0.774
MONA^b^	19	(27)	4	(31)	10	(29)	1.000
CPR^c^	4	(6)	0	(0)	0	(0)	0.317
Single treatments^d^							
Nitro-glycerine	17	(24)	3	(23)	9	(26)	0.817
Morphine	20	(29)	4	(31)	13	(37)	0.385
O_2_ on mask	16	(23)	0	(0)	3	(9)	0.181
O_2_ nose catheter	31	(44)	7	(54)	16	(46)	1.000
Drugs in nebulizer	3	(4)	0	(0)	0	(0)	0.555
Ringer acetate	18	(26)	2	(15)	1	(3)	0.007
Acetylsalicylic acid	17	(24)	4	(31)	10	(29)	0.654
Clopidogrel	12	(17)	0	(0)	7	(20)	0.423
Metoclopramide	22	(31)	4	(31)	11	(31)	1.000
Neck collar	5	(7)	1	(8)	4	(11)	0.476
Data missing	1	(1)	0	(0)	1	(3)	

Comparison between GP involved and GP not involved using Fischer’s exact test. Fisher’s exact test was used for statistical analysis of GP involvement. Multiple treatments are possible.

^a^Includes call-outs and consultation at GP’s office or out-of-hour service clinic.

^b^MONA: morphine, oxygen, nitro-glycerine, acetylsalicylic acid was ordinated.

^c^Cardiopulmonary resuscitation.

^d^One or more treatment was given.

## Results

HEMS Førde and SAR Florø completed 2310 HEMS missions during the study period. We identified 627 cancelled missions. Among these, 73% (455/627) were excluded from our analysis because the missions were completed by neighboring HEMS (33%), there was no longer medical indication for HEMS (20%), or other reasons, such as misclassification or duplicates (20%). Ultimately, the study included a total of 172 missions with 180 patients.

The main reason for mission cancellation was weather conditions, which were reported in nearly 90% of primary and secondary missions. A total of 74% of the cancelled missions were during the six months from October through March, and 46% of the cancelled missions were rejected or aborted during the afternoon (16:00 to 23:59) (not in table).

Table 1 presents demographic data for the 180 included patients, along with mission sites and destinations. Median patient age was 59 years (IQR 31–72). Based on the Index, 73% of the missions were considered acute, 26% urgent, and 1% non-urgent (not in table). The most common diagnostic category was cardiology (35%), followed by neurology (19%) and trauma (15%). Two-thirds were primary missions. When necessary, all patients were transported to a final destination via ground transportation and/or boat ambulance (not in table).

**Figure 1.**
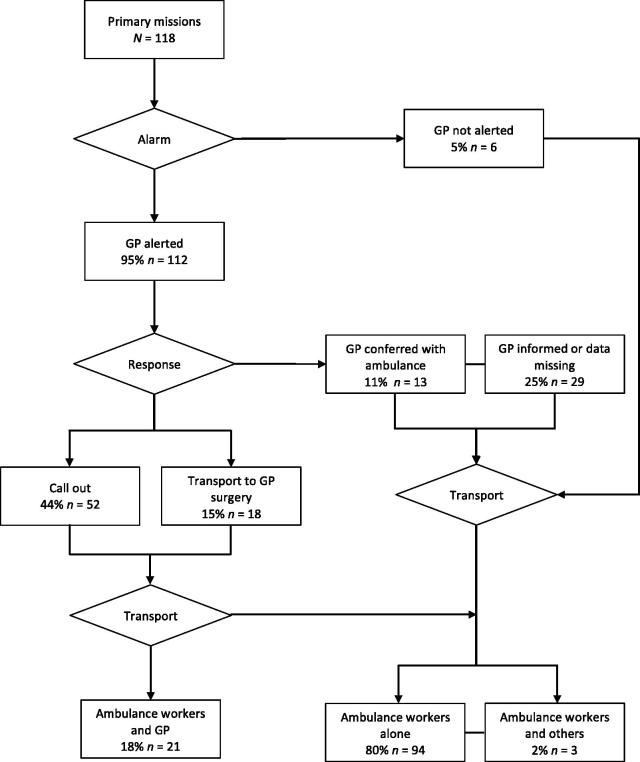
Flow chart of primary mission, showing alerts, GP responses, and transport options.

Among the primary missions, the on-call GP was alerted for 95% (112/118) of the patients (Figure 1). For 46% (52/112) of these patients, the GP responded by calling out, while 16% (18/112) of these patients were transported by ground ambulance to the GP’s office or OOH casualty clinic. In total, 63% (70/112) of the patients were examined by a GP. For 12% (13/112) of these patients, the GP gave advice by telephone to ambulance workers. GP involvement did not differ according to time of day (*p* = 0.601), diagnostic group (*p* = 0.309), or patient’s age (*p* = 0.490). The patient’s final destination did not differ in accordance with GP involvement (*p* = 0.410) (not in table). Among the 52 patients who were examined by a GP on scene, 21 (40%) were accompanied by the doctor during transport to their destination. None of the patients who were primarily localized at a GP’s office or a casualty clinic at the time of HEMS request were accompanied by a GP during transport.

Among the secondary missions (Table 1), 16% (10/62) of the patients were accompanied by a physician together with ambulance workers during transport between hospitals. In 13% (8/62) of cancelled HEMS missions, the patient ultimately stayed at their initial hospital instead of being transported by ground ambulance to a hospital with higher level of competence/care.

Among the patients in primary missions, 26% (31/118) did not receive treatment while 14% (17/118) were provided oxygen (Table 2). GP involvement did not influence the provision of treatment, with the exception of i.v. Ringer acetate. Table 3 shows which health personnel were responsible for the treatment provided to patients in primary missions. In secondary missions, 31% (19/62) of the patients received no treatment during transport. Four patients were intubated before transport, and two patients received continuation of non-invasive ventilation; these patients were accompanied by a physician or nurse anaesthetist (not in table).

**Table 3.** Treatment provided by health personnel group in primary missions

	Primary missions (*N* = 118)	
Health personnel	*N*	%
Ambulance workers	11	(9)
General practitioner	83	(69)
Hospital physician	7	(6)
HEMS anaesthesiologist	9	(8)
Other	2	(2)
Missing	8	(6)

A physician was considered responsible if on scene or after telephone conferral with ambulance workers.

Ambulance workers can administer certain treatments that are pre-delegated by a physician when protocol criteria are fulfilled.

## Discussion

### Principal findings

In this study, we investigated how patients with anticipated need of HEMS are handled when the HEMS mission is cancelled in a rural county in Norway. Many of the patients were provided minimal or no treatment. The on-call GP was alerted in almost all primary missions, and responded with a call-out for nearly half of these patients. The treatment provided in primary missions did not differ according to GP involvement. Many missions were completed by ambulance workers without a physician on site. A physician accompanied the patient during transport in only 17% of the missions; however, most patients received treatment after ambulance workers had conferred with a physician. The most common diagnostic categories were cardiology, neurology, and trauma.

### Strengths and limitations

One strength of this study is that it included all available registered patients over a four-year period, thus providing a good representation of how challenging missions are solved in a rural part of Norway. The analysed patient sample was selected based on the reason for HEMS cancellation, regardless of clinical information. No changes in use of HEMS or organizational changes in the prehospital services has been reported since the study period.

Our present results should be interpreted with some caution, and may not be generalizable to other regions. Notably, there is no national consensus among EMCC areas regarding strict HEMS dispatch criteria, and HEMS responses differ between the bases. For example, in 2014, HEMS Bergen rejected 30% of the missions while HEMS Lørenskog rejected 20% [20]. Compared to other bases, HEMS Førde has a higher rate of completed missions [7], indicating a lower threshold for HEMS usage in this area. Moreover, the proportion of trauma patients in our present study (15%) differs from the proportion among completed HEMS missions on the west coast of Norway (30%) [10], which may indicate different response thresholds depending on the medical indication. It is also possible that the pilot’s decision to reject a mission may have been influenced by the anticipated severity of the patient’s condition and the experience of the crew. Still, our findings are relevant in rural areas abroad Norway with similar prehospital services. Finally, missing information from the databases was search for in several sources. However, the study design and validity of the databases is a potential information bias.

### Findings in relation to other studies

The advantages of HEMS are controversial, and it remains unclear which elements provided by HEMS benefit the patients [19]. However, several studies have reported advantages of HEMS for trauma patients [11–15]. Norwegian studies involving both medical and trauma patients report inconsistent results regarding benefits. One study demonstrated gained life years among patients treated by an anaesthesiologist [16], while another showed that two-thirds of severely ill or injured patients received advanced treatment [10]. However, other studies have concluded that the majority of patients did not receive medical treatment requiring an anaesthesiologist, and thus could thus have been transported by ground ambulance [17,18]. Our findings may indicate the same, though it is difficult to compare the results of different studies due to the difference among study areas, methodological variations, and challenges intrinsic to RCTs in emergency medicine research. Although our present study has some of the same limitations, it contributes to the scarce body of knowledge about cancelled HEMS missions.

In the primary missions in our study, the patients were transported and cared for by ambulance workers, confirming the important role of ground ambulance services in Norway. Moreover, the high proportions of alerted on-call GPs, call-outs by GPs, and telephone conferences between ambulance workers and GPs indicates good collaboration between the OOH and ground ambulance services, as intended by national regulations. Compared with our present findings, a Norwegian study from 2010 reported a lower overall rate of alerting the on-call GP (47%), noting that it was less common for the GP to be alerted when the event was not life threatening [23,24]. In recent years, there has been increased focus on physician attendance in the Norwegian prehospital system. However, we still found in our study that the on-call GP decided not to see the patient in 40% of the cases where the GP was alerted. This was likely due to long time that it would take for the GP to travel to the patient in many cases. Few studies have examined the effects of GP attendance in acute situations [25]; however, Norwegian legislation imposes GPs to call-out when needed [2], ambulance workers prefer GPs to be present in challenging prehospital emergencies [26] and GPs claim improved patient care when involved [27]. Anticipated reasons for low involvement of medical professionals other than ambulance workers during patient transport can include a stable condition of the patient, a less severe medical problem than initial indicated, or a concurrent conflict for the GP. Still, it is notable that no patient had a GP present during ambulance transport in the cases where HEMS was requested to an OOH office or GP office. These patients were considered in need of rapid transport and/or attendance of a HEMS physician. However, when HEMS was not accessible, the patients were transported by ground ambulance with only ambulance workers and with a prolonged transport time to the hospital. Moreover, the same pattern was observed for physician involvement in secondary missions, even though severe clinical conditions might be anticipated in such cases. In secondary missions, only one in six patients was accompanied by a specialist doctor from the requesting hospital.

In primary missions, two-thirds of patients received no specific intervention, or only received treatment that ambulance workers in Norway can administer without physician guidance. Even so, in eight of ten cases, the treatment was ordinated by a physician either on site or by telephone, indicating cooperation between the different prehospital services in the treatment of most acute patients outside of the hospital. For secondary missions, there was a similarly low volume of interventions or treatments. Although few patients received advanced treatment, our experience is that clinical observation and monitoring of patients in potentially life-threatening situations is vitally important. Stroke is one example of an acute situation that requires fast and correct diagnosis with minimal pre-hospital treatment. There is scarce evidence for which situations continuous observation by a physician is required and further research is needed [25,27]. Still, attendance by physicians is probably improving the quality of the patient assessment. The presently reported high proportion of patients receiving minimal treatment is similar to the findings of older studies in Norway [17,28]. However, a recent study of HEMS in Norway reported a high rate of advanced treatment provided by an experienced anaesthesiologist [10]. Procedures performed by an anaesthesiologist through HEMS are not directly comparable to procedures performed by ambulance workers or a GP, and it is not known whether an HEMS anaesthesiologist would have performed different interventions for the patients in our present study.

In primary missions, the patients were most often admitted to a hospital, with only a few patients left on-scene or at the GP’s office. Assuming that hospitalization indicates a need for advanced care, this supports that the initial level of response at the EMCC was appropriate in most cases. Some patients who were considered to require interhospital transfer to a higher level of care ultimately remained at the initial local or county hospital. In these cases, the option to transport the patient via ground ambulance deemed less acceptable than the care provided at the initial hospital. While a local hospital can provide advanced care, critically ill patients may need more intensive care or surgery at another hospital and may therefore want HEMS to transport them. When HEMS was unavailable, some patients remained at their initial hospital. Intubated patients and patients with other advanced treatments who were transported by ambulance were all accompanied by specially trained nurses or physicians in addition to ambulance workers.

## Conclusions

The present results showed that ambulance workers and on-call GPs have important roles when HEMS are unavailable. Our findings indicated good collaboration among the prehospital services. The majority of patient were examined by a GP or cared for by ambulance workers who conferred with a physician. Few patients received advanced treatment, and treatment did not differ according to GP involvement.
